# Model-based conceptualization of thyroid hormone equilibrium via set point and stability behavior

**DOI:** 10.1007/s00285-024-02176-8

**Published:** 2024-12-18

**Authors:** Corinna Modiz, Andreas Körner

**Affiliations:** Institute of Analysis and Scientific Computing, TU Wien, Wiedner Hauptstraße 8, 1040 Vienna, Austria

**Keywords:** HPT complex, Set point determination, Global stability analysis, Mathematical modeling

## Abstract

The HPT complex, consisting of the hypothalamus, pituitary and thyroid, functions as a regulated system controlled by the respective hormones. This system maintains an intrinsic equilibrium, called the set point, which is unique to each individual. In order to optimize the treatment of thyroid patients and understand the dynamics of the system, a validated theoretical representation of this set point is required. Therefore, the research field of mathematical modeling of the HPT complex is significant as it provides insights into the interactions between hormones and the determination of this endogenous equilibrium. In literature, two mathematical approaches are presented for the theoretical determination of the set point in addition to a time-dependent model. The two approaches are based on the maximum curvature of the pituitary response function and the optimal gain factor in the representation of the HPT complex as a closed feedback system. This paper demonstrates that all hormone curves described by the model converge to the derived set point with increasing time. This result establishes a clear correlation between the physiological equilibrium described by the set point and the mathematical equilibrium with respect to autonomous systems of differential equations. It thus substantiates the validity of the theoretical set point approaches.

## Introduction

The thyroid gland plays a pivotal role in regulating metabolism, impacting cardiovascular activity, fat metabolism, and energy usage, among other functions. According to Madariaga et al. (2014), around 11% of Europeans experience thyroid dysfunction. When this system malfunctions, it disrupts hormonal balance, leading to symptoms like fatigue, depression, and weight fluctuations, varying based on the specific thyroid disorder.

The majority of thyroid issues involve either an excess (hyperthyroidism) or a deficiency (hypothyroidism) of hormone production. While synthetic hormones can improve these conditions, determining the right dosage often necessitates multiple doctoral appointments and hormone level assessments. Even when patients fall within the healthy hormone range with medication, their well-being isn’t consistently ensured due to the personalized nature of thyroid regulation and the variability in drug therapy outcomes.

The understanding of this complicated system is still incomplete, which emphasizes the need for descriptive mathematical models in order to gain a better insight into the complexity of the system. Simulating hormone dynamics over time allows us to better understand how different components influence an individual’s physiological balance. This knowledge could potentially lead to more personalized drug dosages, reducing medical visits and enhancing patient well-being.

**Fig. 1:**
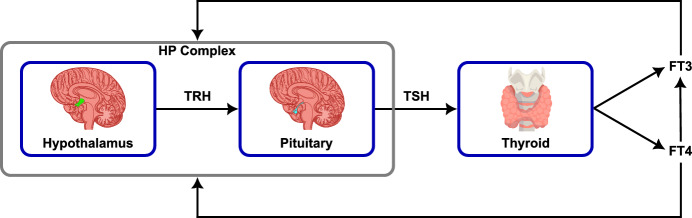
Block diagram of a general closed-loop HPT feedback system including HP complex and thyroid

The thyroid operates together with the pituitary and hypothalamus as part of the Hypothalamus-Pituitary-Thyroid (HPT) complex. The hypothalamus releases thyrotropin-releasing hormone (TRH), stimulating the pituitary to release thyroid-stimulating hormone (TSH), which triggers increased production of thyroid hormones, specifically free triiodothyronine (FT3) and free thyroxine (FT4). This initiates a feedback loop, decreasing TSH and TRH levels. Both FT3 and FT4 show a negative feedback on the HP complex and are converted into each other at intracellular and peripheral level following biochemical processes, see Feldt-Rasmussen et al. (2021). However, clinicians use FT4 and TSH for diagnosis of diseases as, at a macroscopic level, FT4 controls the overall dynamics due to the conversion at different levels. The HPT dynamics are illustrated in Fig. 1.

This work focuses on analyzing a mathematical model describing the dynamics of the HPT complex and introducing a theoretical approach to determine the individual hormonal equilibrium. Various models of different complexities are available in the literature. For instance, the Michaelis-Menten-Hill kinetics, proposed by Dietrich et al. (2004), forms the basis for physiologically plausible pituitary models and is relevant in the HPT context, as established by Frank (2013) and Spencer et al. (1990). Thus, approaches aiming to depict the time-dependent dynamics of the HPT complex often rely on the Michaelis-Menten equation, e.g. Pandiyan et al. (2014); Goede (2021); Yang et al. (2021). More intricate models, as presented in Liu et al. (1994); Martins and Monteiro (2010); Eisenberg et al. (2008), incorporate up to 15 differential equations, consider additional factors such as other hormones than FT4 and TSH and subsystems like kidneys or the peripheral vascular system. However, these more complex models, tailored to specific thyroid conditions, heavily rely on collecting and measuring patient-specific hormonal data. Unfortunately, this detailed data often is not readily available in standard patient datasets, making it challenging to validate these models.

This work focuses on a mathematical model presented in Goede (2021) introducing theoretical approaches to explicitly determine the individual hormonal equilibrium, the so-called set point. It describes the HPT dynamics based on an autonomous two-dimensional system of ordinary differential equations determining the hormonal course of TSH and FT4. This reduction is based on combining hypothalamus and pituitary to the so-called Hypothalamus-Pituitary (HP) complex as TRH secretion is only affected to a small extend by thyroid hormones according to Paschke (2019). Additionally, as described in Silbernagl and Despopoulos (1991), most of FT3 concentration originates from alteration of FT4.

In previous publications (Goede et al. 2014; Leow and Goede 2014), two mathematical approaches for an explicit set point determination were introduced for the response functions of the pituitary and thyroid. It is proposed, that both maximum curvature theory and gain factor analysis result in a theoretical, patient-specific representation of the individual physiological equilibrium. Additionally, a time-dependent model, for which the HP- and thyroid (T)-function represent the equilibrium functions, was presented based on these findings in Goede (2021). The model is a simplified representation of the HPT dynamics and focuses on introducing a time dependency to the HP- and T-function that have been validated extensively in Goede et al. (2014); Leow and Goede (2014).

In this paper, the applicability of the two set point approaches is examined mathematically and subjected to a qualitative analysis. In previous publications on this model, assumptions are made that have not yet been mathematically analyzed and proven, which is precisely demonstrated in this paper. In the event of deviations from the equilibrium, the endogenous control system regulates the thyroid hormones back to the balance point. Therefore, this paper analyzes the long-term behavior of the model trajectories to demonstrate that they converge to the set point based on a global stability analysis. This further substantiates the validity of those mathematical approaches to determine a physiological equilibrium. This analysis will focus in particular on the hypothesis as to what extent these set point theories are related to a mathematical equilibrium of the HPT model.

## Mathematical derivation of the set point theory

The HPT complex strongly depends on the mutual influence of its components and contains an individual hormonal equilibrium, the so-called set point, which is defined as follows.

### Definition 1

The set point of an intact HPT axis of a healthy euthyroid person represents the ideal personalized thyroid function target that results in an optimal healthy state. Leow and Goede (2014).

According to Goede et al. (2014); Leow (2007); Leow and Goede (2014), this equilibrium can be mathematically derived and further investigated by introducing the maximum curvature theory, in which the Leow-Goede equations represent the fundamental law governing the homeostatic euthyroid set point equilibrium in the HPT axis. Additionally, it can be defined by representing the HPT complex as a feedback system, which also provides the possibility to gain further information about the physiological behavior. The two approaches are presented in detail in Goede et al. (2014); Leow and Goede (2014) and summarized in the following.

### Maximum curvature theory (MCT)

Even the slightest deviation from the set point is registered by the HP complex. In response, it tries to regulate the hormones towards the original state of equilibrium, the so-called set point. Thus, the set point is defined as the point of maximum sensitivity of the HP complex, which mathematically corresponds to the point of maximum curvature of the pituitary response function, according to Leow and Goede (2014).

#### Definition 2

The set point of the HPT complex is specified as the unique point of the response function of the pituitary, $f(x) = y$, that fulfills

1 $$\begin{aligned} \frac{\textrm{d}K_f}{\textrm{d}{x}} = 0\quad \text {and} \quad \frac{\mathrm{d^2}K_f}{\textrm{d}{x^2}} < 0 \quad \text {with} \quad K_f = \frac{\frac{\mathrm{d^2 }f}{\textrm{d } x^2}}{\left( 1 + \left( \frac{\textrm{d }f}{\textrm{d }x} \right) ^2 \right) ^{\frac{3}{2}}}. \end{aligned}$$

### Gain factor analysis (GF)

**Fig. 2:**
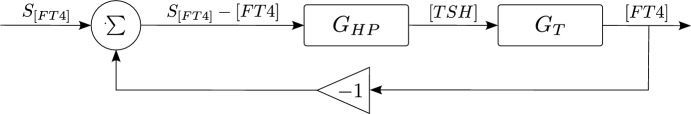
HPT complex represented as negative feedback closed-loop system

The second approach to determine the set point is also discussed in Leow and Goede (2014) and Goede et al. (2014). The HPT complex can be defined as closed loop system including a negative feedback loop and the set point $S_{ \left[ \textrm{FT}{4}\right] }$ as input reference value. An illustration of this system is given in Fig. 2.

The set point definition is based on the loop transfer function *G*(*s*) of the entire system, obtained by breaking the feedback loop and the computation of the corresponding zero frequency gain. The detailed approach can be found in Aström and Murray (2012) and Goede et al. (2014). The loop gain of the HPT complex is therefore defined as $G=|G_{HP}G_{T}|$. According to Goede et al. (2014), when referring to the HPT complex, the interpretation refers to the measure of variation of the output signal to a corresponding relatively small variation of the input signal. Thus, the respective gain factors of the compartments are defined as

2 $$\begin{aligned} G_{HP} = \frac{\textrm{d}{ \left[ \textrm{TSH}\right] }}{\textrm{d}{ \left[ \textrm{FT}{4}\right] }} , \quad G_{T} = \frac{\textrm{d}{ \left[ \textrm{FT}{4}\right] }}{\textrm{d}{ \left[ \textrm{TSH}\right] }} . \end{aligned}$$

Following this framework, the set point can be defined as follows according to Goede et al. (2014).

#### Definition 3

The set point of the HPT complex represented as negative feedback closed-loop system, as depicted in Fig. 2, is defined as the point where the loop gain operates at its optimum. It corresponds to the maximum of *G* depending only on $\left[ \textrm{TSH}\right]$ and thus the point that fulfills

3 $$\begin{aligned} \frac{\textrm{d}G}{\textrm{d}{ \left[ \textrm{TSH}\right] }} = 0\quad \text {and} \quad \frac{\mathrm{d^2}G}{\textrm{d}{ \left[ \textrm{TSH}\right] ^2}} < 0 \quad \text {with} \quad G = |G_T G_{HP}|. \end{aligned}$$

The dependence of *G* solely on $\left[ \textrm{TSH}\right]$ originates from its explicit calculation presented in Goede et al. (2014) with respect to the mathematical model discussed in the following work.

## HPT modeling and set point

The so-called minimal model of the HPT complex, presented in Goede (2021), describes the mutual influence and time-dependent dynamics of both hormones $\left[ \textrm{FT}{4}\right]$ and $\left[ \textrm{TSH}\right]$ as a two-dimensional autonomous system of ordinary differential equations. For reasons of readability, $\left[ \textrm{FT}{4}\right]$ and $\left[ \textrm{TSH}\right]$ are referred to hereinafter as $x = \left[ \textrm{FT}{4}\right]$ and $y = \left[ \textrm{TSH}\right]$ respectively. The model is defined as

4 $$\begin{aligned} \begin{aligned} \frac{\textrm{d}{y}}{\textrm{d}t}&= \frac{S}{\exp (\varphi x)}-y, \\ \frac{\textrm{d}{x}}{\textrm{d}t}&= A - \frac{A}{\exp (\alpha y)}-x. \end{aligned} \end{aligned}$$

The system includes four parameters $S, \varphi , A, \alpha \in \mathbbm {R^+}$, where $\varphi$ and $\alpha$ represent the respective decay rate, *S* and *A*, however, are not associated with physiological values. It should be noted that no additional parameters were included in the equations to take into account the clearance rate of the two hormones. They have not been introduced for the sake of simplicity and readability, as the theoretical results presented in the following chapters can be conducted analogously if this detail is amended. The time-dependent model is established based on findings on the equilibrium function of *y*, the so-called HP-function, for which the maximum curvature theory was originally introduced in Leow and Goede (2014). The HP-function represents the response of the HP complex given a certain concentration of *x*. This negative exponential relationship between *y* and *x* is presented and validated in Goede and Leow (2013) and Leow (2007). The equilibrium function of *x*, the so-called T-function, on the other hand defines the thyroid response function. The HP- and T-function are derived from the equilibrium state of the system, $(\dot{x}, \dot{y}) = (0,0)$. It follows that

5 $$\begin{aligned} y&= \frac{S}{\exp (\varphi x)}, \end{aligned}$$

6 $$\begin{aligned} x&= A - \frac{A}{\exp (\alpha y)}. \end{aligned}$$

To compute the set point, Definition 2 and 3 can be applied. The results for the HP-function, referred to as maximum curvature of hypothalamus-pituitary (MCHP), are already presented in Leow and Goede (2014), and thus are concluded in Table 1. However, the maximum curvature theory should hold true for the T-function since the thyroid also reacts most sensitively to changes in *y* at the set point, as it is defined as the individual hormonal equilibrium that should be ultimately adjusted by the body. The following paragraph shows the agreement with the given definition by demonstrating the theoretical applicability of the maximum curvature theory to the thyroid response function. Importantly, the resulting set point coordinates should not contradict previously published results. By applying Definition 2 to the T-function,, denoted by maximum curvature of thyroid (MCT), the respective curvature function is derived as

7 $$\begin{aligned} K_{x} = \frac{-A \alpha ^2 \exp (-\alpha y)}{\left( 1 + A^2 \alpha ^2\exp (-2 \alpha y) \right) ^{\frac{3}{2}}}, \end{aligned}$$

whereas the respective set point is then given as

8 $$\begin{aligned} \left( x_{sp}^{\text {mct}}, y_{sp}^{\text {mct}}\right) = \left( A - \frac{1}{\sqrt{2}\alpha }, \frac{\ln (\sqrt{2}A\alpha )}{\alpha } \right) . \end{aligned}$$

**Fig. 3:**
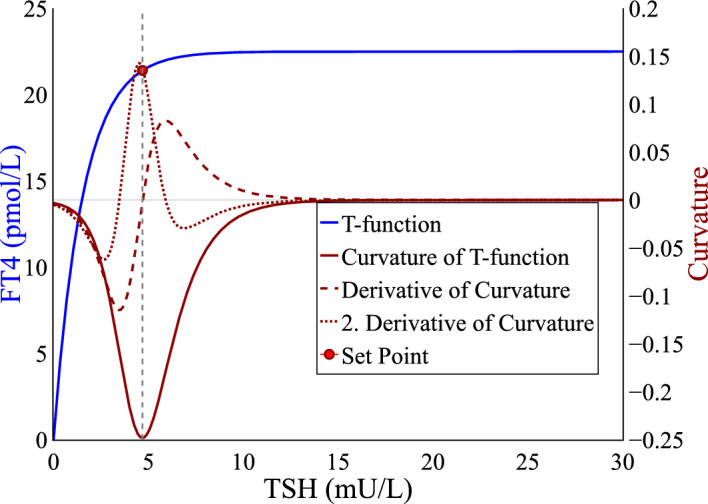
T-function and set point computed based on the corresponding curvature function

This approach therefore also leads to a unique, explicit representation of the set point values of *x* and *y*. The theoretical applicability of the theory of maximum curvature to a function other than the one originally specified can be recognized whilst it nonetheless remains in the context of its objective, the set point determination. The approach is illustrated in Fig. 3.

The set points computed with different methods are summarized in Table 1.

**Table 1:** Application and results of maximum curvature theory and gain factor analysis

Approach	Objective function	Method	$x_{sp}$	$y_{sp}$
MCHP	$K_{y} = \frac{S \varphi ^2 \exp (-\varphi x)}{\left( 1- S^2\varphi ^2\exp (-2 S \varphi x) \right) ^{\frac{3}{2}}}$	$\frac{\textrm{d} K_y}{\textrm{d} x} = 0$	$\frac{\ln (\sqrt{2} S \varphi ) }{\varphi }$	$\frac{1}{\sqrt{2}\varphi }$
MCT	$K_{x} = \frac{-A \alpha ^2 \exp (-\alpha y)}{\left( 1 + A^2 \alpha ^2\exp (-2 \alpha y) \right) ^{\frac{3}{2}}}$	$\frac{\textrm{d} K_x}{\textrm{d} y} = 0$	$A - \frac{1}{\sqrt{2}\alpha }$	$\frac{\ln (\sqrt{2}A\alpha )}{\alpha }$
GF	$G(x,y) = G(y) = A \alpha \varphi y \exp (-\alpha y)$	$\frac{\textrm{d} G}{\textrm{d} y} = 0$	$A \left( 1 - \exp (-1) \right)$	$\frac{1}{\alpha }$

It is apparent that the set point coordinates of both maximum curvature theory applied to the T-function and gain factor analysis depend solely on *A* and $\alpha$.

In line with the underlying theories, it is evident by inserting the respective terms whereby the superscripts ’gf’ and ’mct’ and subscript ’sp’ represent gain function, maximum curvature of thyroid and set point respectively, such that

9 $$\begin{aligned} x_{sp}^{\text {gf}}= x_{sp}^{\text {mct}}\wedge y_{sp}^{\text {gf}}= y_{sp}^{\text {mct}}\quad \Leftrightarrow \quad \sqrt{2}A\alpha = \exp (1). \end{aligned}$$

This suggests that the new application of maximum curvature theory presented in this paper is not in conflict with previously published results, subject to a parameter assumption.

## Qualitative analysis

Since the set point is defined as a physiological equilibrium and the minimal model consists of an autonomous system of differential equations, contextualization with the mathematical equilibrium is possible with regard to stability analysis.

### Local stability behavior

The stability of an autonomous system $\dot{u} = f(u)$ around an equilibrium point can be analyzed by linearization of the respective system using the Taylor series expansion of first order around the equilibrium $u^*$ as development point. In line with Heuser (1991), equilibrium points of a 2-dimensional system of differential equations can then be classified according to the eigenvalues $\lambda _{1,2}$ of the Jacobian evaluated at the equilibrium, ${\textbf {J}} (u^*) \in \mathbbm {R}^{2\times 2}$.

The general derivation of equilibrium points of the minimum model by determining *x* and *y* such that $\dot{x} =0$ and $\dot{y}=0$ leads to equations that cannot be solved analytically due to nested functions as given in

10 $$\begin{aligned} y^*&= S \exp (- \varphi x^*), \nonumber \\ x^*&= A \left( 1 - \frac{1}{\exp (\alpha y^*))} \right) = A \left( 1 - \frac{1}{\exp (\alpha S\exp (-\varphi x^*))} \right) . \end{aligned}$$

**Fig. 4:**
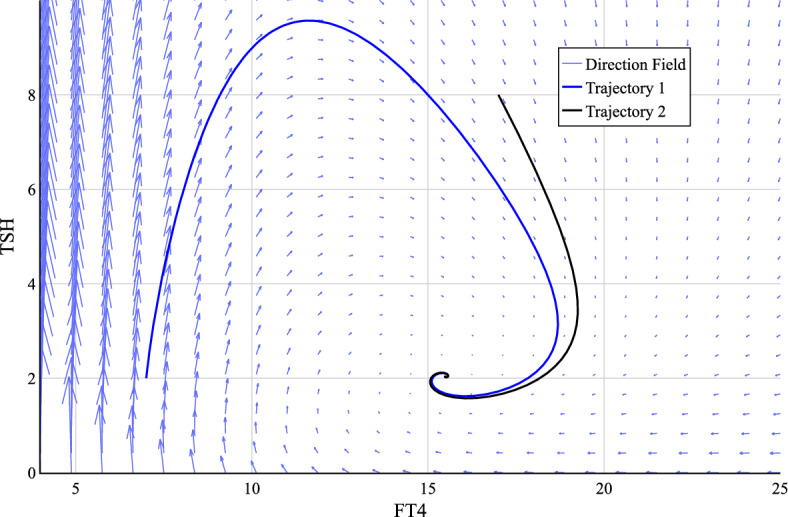
Direction field of model (4) including two sample trajectories. The exemplary parameter set was chosen to be $[S,\varphi , A, \alpha ]=[1000,0.4, 22,0.6]$. The initial value for trajectory 1 is [7, 2] and for trajectory 2 it is [17, 8]

Both maximum curvature theory and gain factor analysis propose the explicit determination of the set point as an equilibrium point. In addition, the directional field of the minimal model, shown in Fig. 4, shows the existence of an asymptotically stable equilibrium point. Combined, this motivates an alternative approach, as carried out in the following.

The combination of the set point coordinates resulting from two approaches (’mchp’ in superscript representing maximum curvature of hypothalamus-pituitary), $(x_{sp}^{\text {mchp}}, y_{sp}^{\text {mchp}})$ and $(x_{sp}^{\text {gf}}, y_{sp}^{\text {gf}})$, is an equilibrium point of the minimal model according to

11 $$\begin{aligned} \frac{\textrm{d}x}{\textrm{d}t}&= A - \frac{A}{\exp (\alpha y_{sp}^{\text {gf}})}-x_{sp}^{\text {gf}}= A \left( 1 - \exp (-1)\right) - A \left( 1 - \exp (-1)\right) = 0, \nonumber \\ \frac{\textrm{d}y}{\textrm{d}t}&= \frac{S}{\exp (\varphi x_{sp}^{\text {mchp}})}-y_{sp}^{\text {mchp}}= \frac{S}{\exp (\ln (\sqrt{2}S\varphi ))} - \frac{1}{\sqrt{2}\varphi } = 0. \end{aligned}$$

Due to the parameter dependencies, not only one derivation of the set point can be selected, but they must be linked. The corresponding Jacobian matrix evaluated at the set point can be derived as

12 $$\begin{aligned} {\textbf {J}}(x_{sp}^{\text {mchp}}, y_{sp}^{\text {gf}}) = \begin{pmatrix} -1 & A\alpha \exp (-\alpha y) \\ S\varphi \exp (-\varphi x) & -1\end{pmatrix}\bigg |_{(x_{sp}^{\text {mchp}}, y_{sp}^{\text {gf}})} = \begin{pmatrix} -1 & A\alpha \exp (-1) \\ -\sqrt{2} & -1\end{pmatrix}. \end{aligned}$$

Provided that $A, \alpha > 0$, the respective eigenvalues can be classified as

13 $$\begin{aligned} \lambda _{1,2} = -1 \pm&\sqrt{- \alpha A \sqrt{2} \exp (-1)} = -1 \pm \textrm{i} \sqrt{ \alpha A \sqrt{2} \exp (-1)} \in \mathbbm {C}. \end{aligned}$$

As $\operatorname {Re}(\lambda _{1,2}) < 0$, this set point is an asymptotically stable spiral sink in line with the direction field shown in Fig. 4.

The set point derived by the maximum curvature theory, applied to the T-function, is also dependent on *A* and $\alpha$. Thus, the combination of $(x_{sp}^{\text {mchp}}, y_{sp}^{\text {mchp}})$ and $(x_{sp}^{\text {mct}}, y_{sp}^{\text {mct}})$ can be shown to be an equilibrium point of the minimal model and analyzed in the same context. An analogous deduction and evaluation of the respective Jacobian matrix results in two complex conjugate eigenvalues with negative real parts and thus an asymptotically stable spiral sink. This further supports that the alternative application of the maximum curvature theory to the T-function results in a representation of the set point as equilibrium point.

Findings on the local stability behavior are expanded to $\mathbbm {R}_+^2$, the total appropriate set for the trajectories as they represent hormone values.

### Global stability behavior

The preceding qualitative analysis proves that the trajectories of the minimal model converge towards the set point with increasing time. This finding is restricted to trajectories found in the neighborhood of the set point as only a local stability analysis was conducted. To extend the results to account for $\mathbbm {R}_+^2$, motivated by Fig. 4 and thus for the entire range of existence of the trajectories, as the hormonal values can only take positive real numbers, the global stability behavior is analyzed. To prove the existence of a global, asymptotically stable equilibrium, it is demonstrated that the Dulac-Bendixon-Criterion and the Poincaré-Bendixon-Theorem apply to the minimal model. The initial procedure is based on the publication (Yang et al. 2021) proving global stability of another mathematical model, which was adjusted accordingly.

#### Proposition 1

The system (4) has no periodic orbits or graphics in $\mathbbm {R}_+^2$.

#### Proof

The set $\mathbbm {R}_+^2$ is open and simply connected and $f_{1,2}$ represent the functions on the right hand side of model (4). Both $f_{1,2}$ are continuously differentiable on $\mathbbm {R}_+^2$. By choosing $D \equiv 1$, it follows that

14 $$\begin{aligned} \frac{\partial (Df_1)}{\partial x} + \frac{\partial (D f_2)}{\partial y} = -2 < 0. \end{aligned}$$

As the term is strictly negative everywhere on $\mathbbm {R}_+^2$, according to the Dulac-Bendixon-Criterion, stated among others in Martcheva (2015), the model has neither periodic orbits nor graphics.

Combined with the next theorem, the main result of this work can be concluded. It is proven by rewriting system (4) that limes inferior and superior of both *x* and *y* are restricted for all time.

#### Theorem 2

All trajectories of the model (4) are constrained in a bounded region of $\mathbbm {R}_+^2$.

#### Proof

The model given in (4) can be rewritten as

15 $$\begin{aligned} \frac{\textrm{d}}{\textrm{d}t} (x+\alpha )&= A - A \textrm{e}^{- \alpha (y+\varphi ) } \textrm{e}^{\alpha \varphi }- (x + \alpha ) + \alpha , \nonumber \\ \frac{\textrm{d}}{\textrm{d}t} ( y + \varphi )&= S \textrm{e}^{-\varphi (x+ \alpha )} \textrm{e}^{\alpha \varphi }-(y+ \varphi ) + \varphi . \end{aligned}$$

Substituting the variables $u = y + \varphi$ and $v = x + \alpha$ results in

16 $$\begin{aligned} \frac{\textrm{d }v}{\textrm{d}t}&= A - A \textrm{e}^{- \alpha u } \textrm{e}^{\alpha \varphi }- v + \alpha , \end{aligned}$$

17 $$\begin{aligned} \frac{\textrm{d }u}{\textrm{d}t}&= S \textrm{e}^{-\varphi v} \textrm{e}^{\alpha \varphi }-u + \varphi . \end{aligned}$$

It will now be shown that $\liminf \limits _{t\rightarrow \infty } v(t) > 0$ and $\liminf \limits _{t\rightarrow \infty } u(t) > 0$ and the limes superior is restricted for both *u*(*t*), *v*(*t*) with respect to $t \rightarrow \infty$.

1. It will be shown that $\limsup \limits _{t\rightarrow \infty } v(t) \le A + \alpha < \infty$. Based on the product rule, equation (16) can be rewritten as
  18 $$\begin{aligned} \frac{\textrm{d }}{\textrm{d}t} (\textrm{e}^t v(t)) = - A \textrm{e}^{- \alpha u + \alpha \varphi + t} + \textrm{e}^t ( A + \alpha ) . \end{aligned}$$
  By integrating the equation with respect to *t*, it follows that
  19 $$\begin{aligned} \textrm{e}^t v(t) - v(0)&= - \int _{0}^{t} \frac{A \textrm{e}^{\alpha \varphi + s}}{\textrm{e}^{\alpha u}} \, \textrm{d}s + \int _{0}^{t} \textrm{e}^s ( A + \alpha ) \, \textrm{d}s \nonumber \\&= - \int _{0}^{t} \frac{A \textrm{e}^{\alpha \varphi + s}}{\textrm{e}^{\alpha u}} \, \textrm{d}s + \textrm{e}^t (A+\alpha ) (1-\textrm{e}^{-t}) \nonumber \\&< \textrm{e}^t (A+\alpha ) (1-\textrm{e}^{-t}). \end{aligned}$$
  Thus,
  20 $$\begin{aligned} v(t) < (A+\alpha ) (1-\textrm{e}^{-t}) + \textrm{e}^{-t} v(0), \end{aligned}$$
  which implies
  21 $$\begin{aligned} v^*:=\limsup \limits _{t\rightarrow \infty } v(t) \le A+\alpha < \infty . \end{aligned}$$
2. Analogously, it can be proven that $\liminf \limits _{t\rightarrow \infty } u(t) \ge \varphi > 0$ by reformulating equation (17) such that
  22 $$\begin{aligned} \frac{\textrm{d }}{\textrm{d}t} (\textrm{e}^t u(t)) = S \textrm{e}^{ - \varphi v + \alpha \varphi + t} + \textrm{e}^t \varphi . \end{aligned}$$
  By again integrating both sides of the equation with respect to *t*, it follows that
  23 $$\begin{aligned} \textrm{e}^t u(t) - u(0)&= \int _{0}^{t} \frac{S \textrm{e}^{\alpha \varphi + s}}{\textrm{e}^{\varphi v}} \, \textrm{d}s + \int _{0}^{t} \textrm{e}^s \varphi \, \textrm{d}s \nonumber \\&> \textrm{e}^t \varphi (1-\textrm{e}^{-t}). \end{aligned}$$
  Therefore,
  24 $$\begin{aligned} u(t) > \varphi (1-\textrm{e}^{-t}) + \textrm{e}^{-t} u(0), \end{aligned}$$
  which finally leads to
  25 $$\begin{aligned} u_*:= \liminf \limits _{t\rightarrow \infty } u(t) \ge \varphi > 0. \end{aligned}$$
3. To show that $v_*:= \liminf \limits _{t\rightarrow \infty } v(t) > 0$, let $O:= \{(v(t),u(t)): 0 \le t < \infty \}$ be an orbit lying in the first quadrant. Let $Z:= \{0 \le t_1< t_2<...< t_n <...\}$ be the sequence of points in $[0,\infty )$ such that $\dot{v}(t_j) = 0$ for $j=1,2,...$. It holds that $\inf _{t\ge 0}v(t) = \min \{ v(0), v(t_1),...,v(t_n),...\}$. Since the equilibrium is a spiral point, *Z* is an infinite set which implies that $(t_n)$ is an increasing sequence tending to $\infty$. Additionally, let there be an $\epsilon > 0$ close to zero such that $\varphi - \epsilon < \varphi \le u_*$. Such an $\epsilon$ exists since $\varphi > 0$. Therefore, let $\bar{u}$ be chosen such that $\varphi - \epsilon< \bar{u} < u_*$ and let $\bar{v}$ be the intersection of the vertical line $u=\bar{u}$ and the hyperbola $v = - A \textrm{e}^{- \alpha u } \textrm{e}^{\alpha \varphi } + A + \alpha$. Since $\bar{u} > \varphi - \epsilon$, it follows that
  26 $$\begin{aligned} \bar{v} = - A \textrm{e}^{- \alpha \bar{u} } \textrm{e}^{\alpha \varphi } + A + \alpha > - A \textrm{e}^{- \alpha (\varphi - \epsilon ) } \textrm{e}^{\alpha \varphi } + A + \alpha = - A \textrm{e}^{ \alpha \epsilon } + A + \alpha \end{aligned}$$
  The parameter $\alpha$ defines the decay rate with respect to equation (16). Therefore, it holds that $\alpha < 1$ and additionally
  27 $$\begin{aligned} \bar{v}> - A \textrm{e}^{ \alpha \epsilon } + A + \alpha \underset{\epsilon \rightarrow 0}{\longrightarrow }\ \alpha > 0. \end{aligned}$$
  Since $\bar{u} < u_*$, there exists an $\bar{t}$ such that $u(t) > \bar{u}$, $\forall t_j \ge \bar{t}$. This implies that the orbit $O_{\bar{t}}:= \{(v(t),u(t)): \bar{t} \le t < \infty \}$ lies on the right hand side of the vertical line $u = \bar{u}$. Therefore it follows that the intersections of $O_{\bar{t}}$ and the hyperbola $v = - A \textrm{e}^{- \alpha u } \textrm{e}^{\alpha \varphi } + A + \alpha$ all have their *v*-coordinates greater than $\bar{v}$. Thus,
  28 $$\begin{aligned} v(t_j) \ge \bar{v} \quad \forall t_j \ge \bar{t}. \end{aligned}$$
  Let $j_0$ be an index chosen such that $t_j \ge \bar{t}$ $\forall j \ge j_0$. Then it can be concluded that
  29 $$\begin{aligned} \inf _{t \ge 0} v(t) = \min { \{ v(0),v(t_1),...,v(t_{j_0}) \} }> \bar{v} > 0. \end{aligned}$$
4. Finally, it can be shown that $\limsup \limits _{t\rightarrow \infty } u(t) < \infty$ by using the already proven properties. Since $v_*> 0$ and $v^*< \infty$, $v_0$ can be chosen such that $0< v_0 < v^*$ is fulfilled. Thus, there exists some $t^*$ such that $\forall t \ge t^*: v_0 < v(t)$. Similar to previous approaches, *u*(*t*) can be estimated following
  30 $$\begin{aligned} \frac{\textrm{d }}{\textrm{d}t} (\textrm{e}^t u(t))&= S \textrm{e}^{ - \varphi v + \alpha \varphi + t} + \textrm{e}^t \varphi \nonumber \\ \Leftrightarrow \textrm{e}^t u(t)&= u(0) + \int _{0}^{t} \frac{S \textrm{e}^{\alpha \varphi + s}}{\textrm{e}^{\varphi v}} \, \textrm{d}s + \int _{0}^{t} \textrm{e}^s \varphi \, \textrm{d}s \nonumber \\ \Leftrightarrow \textrm{e}^t u(t)&= u(0) + \int _{0}^{t^*} \frac{S \textrm{e}^{\alpha \varphi + s}}{\textrm{e}^{\varphi v}} \, \textrm{d}s + \int _{t^*}^{t} \frac{S \textrm{e}^{\alpha \varphi + s}}{\textrm{e}^{\varphi v}} \, \textrm{d}s+ \int _{0}^{t} \textrm{e}^s \varphi \, \textrm{d}s. \end{aligned}$$
  According to the prior considerations, by using $\textrm{e}^{v_0} < \textrm{e}^{v(t)}$ for $t \ge t^*$ since the exponential function is strictly monotonously increasing, *u*(*t*) can be further estimated following
  31 $$\begin{aligned} \textrm{e}^t u(t)&< u(0) + \int _{0}^{t^*} \frac{S \textrm{e}^{\alpha \varphi + s}}{\textrm{e}^{\varphi v}} \, \textrm{d}s + \int _{t^*}^{t} \frac{S \textrm{e}^{\alpha \varphi + s}}{\textrm{e}^{\varphi v_0}} \, \textrm{d}s+ \int _{0}^{t} \textrm{e}^s \varphi \, \textrm{d}s \nonumber \\&= u(0) + \int _{0}^{t^*} \frac{S \textrm{e}^{\alpha \varphi + s}}{\textrm{e}^{\varphi v}} \, \textrm{d}s + S \textrm{e}^t \textrm{e}^{\varphi (\alpha - v_0)} (1 - \textrm{e}^{-t + t^*}) + \textrm{e}^t \varphi (1-\textrm{e}^{-t}). \end{aligned}$$
  Thus,
  32 $$\begin{aligned} u(t)&< \textrm{e}^{-t} u(0) + \textrm{e}^{-t} \underbrace{\int _{0}^{t^*} \frac{S \textrm{e}^{\alpha \varphi + s}}{\textrm{e}^{\varphi v}} \, \textrm{d}s}_{< \infty } + S \textrm{e}^{\varphi (\alpha - v_0)} (1 - \textrm{e}^{-t + t^*}) + \varphi (1-\textrm{e}^{-t}), \end{aligned}$$
  and finally
  33 $$\begin{aligned} \limsup \limits _{t\rightarrow \infty } u(t)&< S \textrm{e}^{\varphi (\alpha - v_0)} (1 - \underbrace{\textrm{e}^{t^*}}_{< \infty }) + \varphi< S \textrm{e}^{\varphi (\alpha - v_0)} + \varphi < \infty . \end{aligned}$$

$$\square $$

The existence of a global asymptotically stable equilibrium can then be proven by combining all of the previous findings resulting in the following Theorem.

#### Theorem 3

Model (4) contains an equilibrium point which is globally asymptotically stable.

#### Proof

It is proven that the set point is an equilibrium point independent of the individually derived set point equations. More specifically, the set point is a locally asymptotically stable spiral point. Theorem 2 proves that all trajectories of model (4) are constrained in a bounded region of $\mathbbm {R}_+^2$ and therefore, according to the Poincaré-Bendixon-Theorem, as stated in Martcheva (2015), the set point is globally asymptotically stable since Proposition 1 rules out periodic orbits and graphics due to the Dulac-Bendixon-Criterion.

It is shown that all physiologically reasonable trajectories in $\mathbbm {R}_+^2$ of the minimal model tend towards the set point with increasing time. The model was established based on its equilibrium equations for which two mathematical approaches were introduced to theoretically derive the set point. Based on this work, it is shown that this set point, defined to represent the individual physiological equilibrium, corresponds to the mathematical equilibrium of the respective autonomous system. As it is global asymptotically stable, all hormone trajectories stabilize at the set point value. This corresponds to physiological behavior as the endogenous balance is restored even after small deviations. Based on this proof, the mathematical validity of both maximum curvature theory and gain factor analysis can be concluded as this concept of the set point provides a possible association of the physiological and mathematical equilibrium.

Additionally, by combining the set point equations derived from different approaches, the autonomous system can be described by only two out of four parameters since the remaining ones can be derived from the others. This approach is presented in the next section alongside parameterized solution curves to contextualize the theoretical findings with patient-specific hormonal courses.

## Patient-specific numerical illustration

Having established a relation between a theoretical representation of the set point derived by two approaches and the mathematical equilibrium of systems of differential equations, the results are illustrated in this section.

This approach also allows for the minimal model to be described with two parameters as the remaining ones can be derived based on the others as presented in the following. If patient data is available, a parameter identification with respect to the HP-function in the state space is sufficient to compute the patient-specific set point according to the previous work. Based on the following procedure, also presented in Leow and Goede (2014), all four parameters $S, \varphi , A$ and $\alpha$ and both set point coordinates can be determined. Having identified both *S* and $\varphi$ in the course of a calibration of the HP-function to patient data in the state space, the respective set point $\left( x_{sp}^{\text {mchp}}, y_{sp}^{\text {mchp}}\right)$ can be computed using the maximum curvature theory. The remaining parameters *A* and $\alpha$ are determined by transforming $\left( x_{sp}^{\text {gf}}, y_{sp}^{\text {gf}}\right)$ given in Table 1, which results in

34 $$\begin{aligned} A = \frac{x_{sp}}{1-\exp (-1)}, \qquad \alpha = \frac{1}{y_{sp}}. \end{aligned}$$

**Fig. 5:**
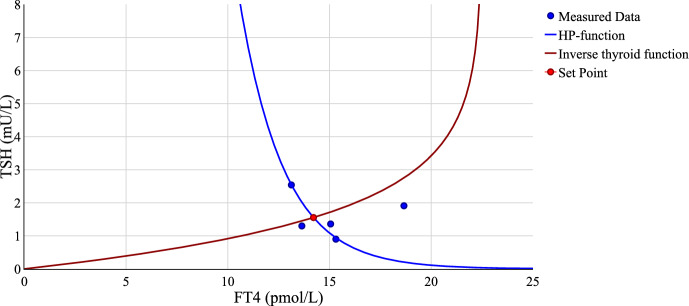
HP- and inverse T-function as response of HP-complex and thyroid in state space including measured patient data. The set point was determined as $(x_{sp}, y_{sp}) = (14.21,1.55)$ with the parameter set $[S, \varphi , A, \alpha ]=[1000.53,0.45,22.49,0.64]$

An exemplary state space plot to illustrate this approach including measurements of one patient is presented in Fig. 5. The patient data was collected at the Vienna General Hospital in the course of a retrospective study conducted by the Medical University of Vienna. To include the thyroid response function in the same plot, the inverse T-function is computed as

35 $$\begin{aligned} y = -\frac{1}{\alpha } \ln \left( \frac{A-x}{A}\right) . \end{aligned}$$

The results are shown in Fig. 5.

As presented in this plot, the value of the HP-function is large if the input is small and vice versa. In the case of the T-function, the secretion of FT4 increases if the TSH value rises as the thyroid gland is stimulated. Thus, the two curves accurately represent the endogenous HPT dynamics. In line with the described approach for parameter identification, the set point corresponds with the point of intersection of both response functions. It can be seen that the set point is close to the mean value of the patient data if the point with the greatest distance to the HP-function is regarded as an outlier. As also stated in Leow and Goede (2014), the HP-curve then accurately represents the data dynamics of this patient.

Since all parameters are already identified, the time-dependent curves of the same patient can be plotted to illustrate previously proven results.

**Fig. 6:**
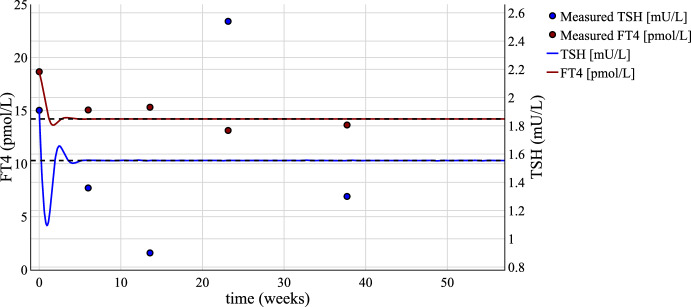
Time-dependent FT4 and TSH curves including respective patient data. The set point $(x_{sp}, y_{sp}) = (14.21,1.55)$ is represented by the dashed line. The parameter set is again $[S, \varphi , A, \alpha ]=[1000.53,0.45,22.49,0.64]$

Fig. 6 shows that the time-dependent curves for both FT4 and TSH level off at the equilibrium after a few weeks and thus illustrates the proven existence of an equilibrium point. Additionally, it has to be noticed that the set point of both hormones, included as dashed lines in the plot, corresponds to this same equilibrium in line with the theoretical findings presented in section 4. Thus, the established relationship between the set point derived by maximum curvature theory and gain factor analysis and the theoretical equilibrium of an autonomous system of differential equations can also be observed with the time-dependent curves.

The time-dependent dynamics of the patient measurements are not accurately represented as the trajectories level off at the equilibrium after a short time span. It has to be mentioned that the original objective of the model was to introduce mathematical approaches to define the set point rather than the time-dependent hormonal course. Nevertheless, the model shows physiologically valid dynamics as FT4 and TSH decrease at first such that FT4 approaches the set point. As TSH rises again, FT4 increases after a time delay before both level off at the set point. Additionally, the last point of the patient data is close to the equilibrium of both curves. For this patient, it is noted in her medical records that she is sufficiently thyroxine-treated. This could imply that her physiological hormonal equilibrium is close to her last measurement, which can be found in close distance to the set point in Fig. 6 as the theoretical equilibrium.

## Discussion

The HPT complex forms a controlled system holding an equilibrium, the set point, that is adjusted even after small variations in hormone concentration. Thus, in this work, a mathematical model introducing theoretical approaches to determine the set point in explicit terms is analyzed and evidence of global stability behavior is published for the first time.

The maximum curvature theory and the gain factor analysis are introduced to derive set point coordinates mathematically. The maximum curvature theory is defined in literature for the HP-function, based on the set point corresponding to the point of maximum sensitivity to any changes of the pituitary. In line with the physiological dynamics, the thyroid is also most sensitive to any variations from the endogenous equilibrium. Therefore, the maximum curvature theory is applied to the response function of the thyroid, the T-function, in this work resulting in set point coordinates in explicit terms. Thus, this finding suggests that the maximum curvature theory is applicable to an alternative function while maintaining its original purpose, the set point determination. The gain factor analysis results in a set point depending on the same two parameters as the maximum curvature theory applied to the T-function. In line with the underlying theories and the set point definition as the individual physiological equilibrium, it is shown in this work that those two results are non-contradictory subject to a parameter condition. This further substantiates the applicability of the maximum curvature theory in the context of theoretically determining the set point.

Both HP- and T-function were originally defined to represent the respective state space dynamics of TSH and FT4. In the most recent publication (Goede 2021), a two-dimensional autonomous system of differential equations was established based on the HP- and T-function, which represent the equilibrium functions of the system. Since the set point is introduced to represent the physiological equilibrium based on these functions, the main result of this work is the mathematical proof that it corresponds to the theoretical equilibrium of the autonomous system of differential equations in the context of stability analysis. The general approach to determine the equilibrium point of the system leads to equations that are not analytically solvable due to nested functions. But in spite of this, an asymptotically stable equilibrium point is discernible in the corresponding direction field. This motivates the approach conducted in this work, where the set point is proven to be an asymptotically stable equilibrium point based on linearization of the system in its neighborhood. As the differential equation for TSH and FT4 depend on different parameters, set point coordinates resulting from two distinct approaches have to be combined for this proof. In accordance with the set point definition, the maximum curvature theory applied to the HP-function in conjunction with the gain factor analysis and the maximum curvature theory applied to the T-function, respectively, lead to asymptotically stable equilibrium points of the system. Since also the combination of both maximum curvature approaches results in the correspondence of set point and equilibrium point, the further applicability of this theory is supported in line with previously discussed results.

As of now, the correspondence of set point determined by different approaches and equilibrium point and its local stability is proven. Motivated by the respective direction field shown in Fig. 4, these results are extended in this work to account for all trajectories found in $\mathbbm {R}_+^2$. These represent all physiologically reasonable solution curves as they describe the hormonal progression. To prove the global stability of the set point, it is shown in this work that the Dulac-Bendixon-Criterion and the Poincaré-Bendixon-Criterion apply to the model. The main mathematical result is the proof of Theorem 2 which shows that all trajectories of the model are bound in $\mathbbm {R}_+^2$ based on a reformulation of the model and the restriction of both limes inferior and superior of the trajectories. By combining all of the results, this work proves that the set point corresponds to a global asymptotically stable equilibrium point and thus all physiologically trajectories tend to it with increasing time. This accurately describes the physiological behavior as the endogenous balance is restored after deviations in an euthyroid individual. To summarize, it is ultimately proven that the set point, defined to represent the individual physiological equilibrium, corresponds to the mathematical equilibrium of the respective autonomous system.

The work is concluded with an illustration of the theoretical results using patient data collected at the Vienna General Hospital in the course of a collaboration with the Medical University of Vienna. Based on the set point equations, the model can be described by two out of four parameters as the remaining ones can be derived using the others. This provides a reduced calibration approach, whereas only the HP-function is fitted to data in state space. In line with the calculations, the HP-function and the T-function then intersect at the set point in state space. Since all of the model parameters are identified, also the time-dependent curves can be illustrated.

The model was originally proposed for thyroid-healthy individuals. In this paper, the data of a patient with hypothyroidism is used for illustration, as only one blood sample is usually taken from healthy patients. Since the target value refers to long-term behavior, this approach is sufficient to show that the time-dependent curves level off at the theoretical set point equations. As the original purpose of the model is the set point determination, the observations with respect to the last points of the solution curves are of interest. As demonstrated, the curves of both TSH and FT4 level off at an equilibrium which corresponds to the respective set point coordinates. Thus, the theoretical results can also be observed with patient-specific, time-dependent curves.

In conclusion, the findings of this work support the proposition that maximum curvature theory and gain factor analysis are mathematically valid approaches to derive the set point as equilibrium point. Based on these findings, the set point is a well-founded concept to gather further information about the patient-specific hormonal equilibrium of the HPT complex. In order to better represent the hormone axis of a patient, other characteristics such as weight and age, which have been shown to influence the HPT complex, are to be included in the model. In addition, a parameter that includes medication must be introduced for people with thyroid disease. Provided this extension, the now well-founded concept of the set point offers a way to make an initial medication suggestion that is better targeted at the individual hormonal balance.

## Data Availability

Patient data used in this work is not publicly available to preserve individuals’ privacy as it concerns medical information.
